# Custard Apple (*Annona squamosa* L.) Leaves: Nutritional Composition, Phytochemical Profile, and Health-Promoting Biological Activities

**DOI:** 10.3390/biom11050614

**Published:** 2021-04-21

**Authors:** Manoj Kumar, Sushil Changan, Maharishi Tomar, Uma Prajapati, Vivek Saurabh, Muzaffar Hasan, Minnu Sasi, Chirag Maheshwari, Surinder Singh, Sangram Dhumal, Radha, Mamta Thakur, Sneh Punia, Varsha Satankar, Ryszard Amarowicz, Mohamed Mekhemar

**Affiliations:** 1 Chemical and Biochemical Processing Division, ICAR—Central Institute for Research on Cotton Technology, Mumbai 400019, India; 2 Division of Crop Physiology, Biochemistry and Post-Harvest Technology, ICAR-Central Potato Research Institute, Shimla 171001, India; Sushil.Changan@icar.gov.in; 3 ICAR—Indian Grassland and Fodder Research Institute, Jhansi 284003, India; maharishi.tomar@icar.gov.in; 4 Division of Food Science and Postharvest Technology, ICAR—Indian Agricultural Research Institute, New Delhi 110012, India; uma_11103@iari.res.in (U.P.); vivek_11593@iari.res.in (V.S.); 5 Agro Produce Processing Division, ICAR—Central Institute of Agricultural Engineering, Bhopal 462038, India; muzaffar.hasan@icar.gov.in; 6 Division of Biochemistry, ICAR—Indian Agricultural Research Institute, New Delhi 110012, India; minnusasi1991@gmail.com; 7 Department of Agriculture Energy and Power, ICAR—Central Institute of Agricultural Engineering, Bhopal 462038, India; chirag.maheshwari@icar.gov.in; 8 Dr. S.S. Bhatnagar University Institute of Chemical Engineering and Technology, Panjab University, Chandigarh 160014, India; ssbhinder@pu.ac.in; 9 Division of Horticulture, RCSM College of Agriculture, Kolhapur 416004, India; sdhumal@msu.edu; 10 School of Biological and Environmental Sciences, Shoolini University of Biotechnology and Management Sciences, Solan 173229, India; radhuchauhan7002@gmail.com (R.); mamtaparmar369@gmail.com (M.T.); 11 Department of Food, Nutrition, & Packaging Sciences, Clemson University, Clemson, SC 29634, USA; snehpunia69@gmail.com; 12 Ginning Training Centre, ICAR—Central Institute for Research on Cotton Technology, Nagpur 440023, India; satankarvarsha@gmail.com; 13 Institute of Animal Reproduction and Food Research, Polish Academy of Sciences, 10-748 Olsztyn, Poland; r.amarowicz@pan.olsztyn.pl; 14 Clinic for Conservative Dentistry and Periodontology, School of Dental Medicine, Christian-Albrecht’s University, 24105 Kiel, Germany

**Keywords:** custard apple leaves, biological activities, phenolic bioactives, health promoting effects

## Abstract

*Annona squamosa* L. (custard apple) belongs to the family Annonaceae and is an important tropical fruit cultivated in the West Indies, South and Central America, Ecuador, Peru, Brazil, India, Mexico, the Bahamas, Bermuda, and Egypt. Leaves of custard apple plants have been studied for their health benefits, which are attributed to a considerable diversity of phytochemicals. These compounds include phenol-based compounds, e.g., proanthocyanidins, comprising 18 different phenolic compounds, mainly alkaloids and flavonoids. Extracts from *Annona squamosa* leaves (ASLs) have been studied for their biological activities, including anticancer, antidiabetic, antioxidant, antimicrobial, antiobesity, lipid-lowering, and hepatoprotective functions. In the current article, we discussed the nutritional and phytochemical diversity of ASLs. Additionally, ASL extracts were discussed with respect to their biological activities, which were established by in vivo and in vitro experiments. A survey of the literature based on the phytochemical profile and health-promoting effects of ASLs showed that they can be used as potential ingredients for the development of pharmaceutical drugs and functional foods. Although there are sufficient findings available from in vitro and in vivo investigations, clinical trials are still needed to determine the exact effects of ASL extracts on human health.

## 1. Introduction

*Annona squamosa* L. (Annonaceae), also known as “custard apple,” is a tropical, endemic species of the West Indies, South and Central America, Ecuador, Peru, Brazil, India, Mexico, Bahamas, Bermuda, and Egypt [1,2,3]. In India, as reported by the Indian Council of Agricultural Research (ICAR), *Annona squamosa* is extensively cultivated in various states (Maharashtra, Gujarat, Madhya Pradesh, Chhattisgarh, Assam, Uttar Pradesh, Bihar, Rajasthan, Andhra Pradesh, and Tamil Nadu) with a total area of 40,000 ha [4]. *Annona squamosa* is known for its edible fruits, and the tree grows as a small sapling, rising from 3 m and reaching up to 8 m, with large, randomly spread branches having brownish or light brownish bark with thin leaves [1]. *Annona squamosa* has been utilised as a natural medicine and in various other food applications, e.g., its pulp is utilised as a flavouring agent in ice cream, and 50–80% of custard apple fruit is edible and can be pulped as juice. It contains appreciable vitamin C in the range of 35–42 mg per 100 g, and dietary fibre, vitamin B1 (thiamine), and potassium contents are also notably high [5].

**Figure 1:**
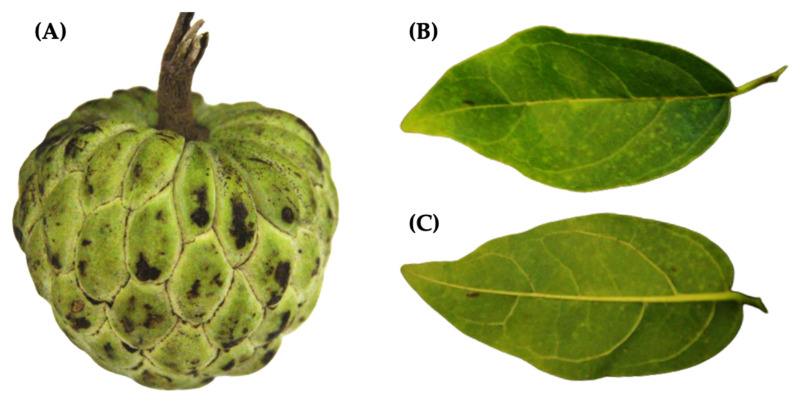
(**A**) *Annona squamosa* L. fruit and leaves. (**B**) Dorsal view of *Annona squamosa* L. leaves. (**C**) Ventral view of *A. squamosa* L. leaves.

Recent articles have demonstrated that various plant byproducts, such as fruit or vegetable pomace, bran/husk/seed coat, seeds, peel, and leaves, are important source of phytochemicals and can be utilised as innovative ingredient in foods [6,7,8,9,10,11,12,13,14,15,16,17]. Extracts obtained from various sections of the *Annona squamosa* plant, such as its bark, roots, leaf, stem, fruit, peel, and seeds, have been utilised in traditional pharmacological applications in different countries to cure a variety of diseases, such as dysentery, epilepsy, haemorrhage, fever, and tumours [2]. *Annona squamosa* seed powder is utilised to abolish lice, leaf extract is used to pacify boils and treat ulcers, and the fruit acts as a sedative in cases involving heart ailments and can be used to alleviate vomiting and treat tumours [18]. Phytochemical studies have revealed that custard apple contains numerous phenol-based compounds, e.g., proanthocyanidins, with 18 different phenolic compounds, mainly alkaloids or flavonoids [19]. Apart from fruit, large amounts of leaves are generated during pruning, which causes complications related to their disposal for farmers. *Annona squamosa* leaves (ASLs) possess valorisation potential owing to their extensive pharmacological properties and biological activities, such as antioxidant, antimicrobial, antidiabetic, antiviral, anticancer, and hepatoprotective activities. *Annona squamosa* fruit and leaf are shown in Figure 1. These activities are caused by the presence of glycosides, phytosterols, carbohydrates, oils, saponins, tannins, alkaloids, phenols, flavonoids, peptides, and various acetogenin compounds [5,18,19,20]. Phytochemical assessments have emphasised that numerous active compounds, such as acetogenins and flavonoids, present in *Annona squamosa* also give rise to plant cytotoxic, antimalarial, antidiabetic, and immunosuppressive activities. Extract of ASLs helps maintain plasma insulin and lipid profiles and can significantly reduce blood glucose and lipid peroxidation [5]. The existing literature on *Annona squamosa* lacks extensive compilation of pivotal information on its phytochemical, nutraceutical, and biological activities. Hence, the present review is a sincere effort to aggregate crucial information regarding the nutritional, pharmacological, and biological aspects and activities of ASLs.

## 2. Nutritional Composition

### 2.1. Protein

*Annona squamosa* is a lowland tropical shrub that possesses a high pharmaceutical potential for treating cardiac ailments, thyroid-related disorders, diabetes, and cancer. Phytochemical analysis of ASL extracts revealed the presence of numerous phytochemicals, such as proteins, carbohydrates, saponins, alkaloids, flavonoids, phenolics, and glycosides [21]. A study conducted at four different sites in Egypt showed the highest protein content in ASLs compared to seeds and fruit. ASLs from Menofia showed a protein content of 13.47 mg/g on a fresh weight (FW) basis, whereas ASLs from Mansoura demonstrated the highest protein content of 17.26 mg/g FW. ASLs from Alexandria (3.52 mg/g FW) and Giza (6.80 mg/g FW) showed the lowest protein content. Thus, a higher protein content in ASL extracts can harness the nutritional value of the food for use by humans as well as animals [22]. Proteins and amino acids were also present in high quantities in the methanolic and aqueous extracts of ASLs [23]. The biuret test confirmed the presence of proteins and amino acids in aqueous ASL extracts, while Millon’s test confirmed the presence of proteins and amino acids in methanolic ASL extracts [24]. Limited research has been conducted to date regarding the quantification of amino acids and protein in ASLs; hence, more attention is needed to explore its protein and amino acid profiles.

### 2.2. Minerals and Vitamins

ASLs possess appreciable concentrations of various minerals, such as phosphorus (P), potassium (K), iron (Fe), calcium (Ca), magnesium (Mg), sodium (Na), copper (Cu), selenium (Se), and zinc (Zn), and vitamins, namely, A, C (ascorbic acid), E, B1 (thiamine), B2 (riboflavin), B3 (niacin), and B9 (folic acid). These minerals are required to maintain a healthy human body, as they help to perform various activities, such as maintenance of healthy teeth and bones, muscle contraction and relaxation, blood clotting, blood pressure regulation, nerve functioning, immune system health, energy metabolism, and many enzymes [25]. Macro- and microminerals, namely, Mg (65.65 mg/100 g), P (43.1 mg/100 g), Zn (0.455 mg/100 g), Cu (0.312 mg/100 g), and Se (in trace amounts), were reported in extracts of ASLs [26]. In another study, Shukry et al. 2019 [22] showed the leaf mineral composition of four different varieties of AS: K (252.47–386.98 μmol/g DW), Na (61.2–95.18 μmol/g DW), Ca (78.51–171.65 μmol/g DW), and Fe (37.23–49.55 μmol/g DW).

The concentrations of vitamins B9 and C were reported to be 8.12–11.98 and 11.98–16.78 μg/g FW, respectively, in ASL [22]. The vitamin contents of the ASL extract showed the presence of vitamin B1 (0.065 mg/100 g), B2 (0.155 mg/100 g), B3 (1.105 mg/100 g), C (34.4 mg/100 g), and A and E (in traces) [26]. HPLC analysis of ASLs revealed a valuable vitamin C concentration of 3.5% [27]. These vitamins are involved in many activities in the human body, such as maintaining skin health, epithelial tissue development, bone development, visual sharpness, immune response, wound healing, and strengthening connective tissues. Vitamin B1, B2, B3, and B9 act as cofactors for several enzymes involved in oxidation-reduction reactions and carbohydrate metabolism [25].

## 3. Phytochemical Profile

### 3.1. Essential Oil Profile

**Figure 2:**
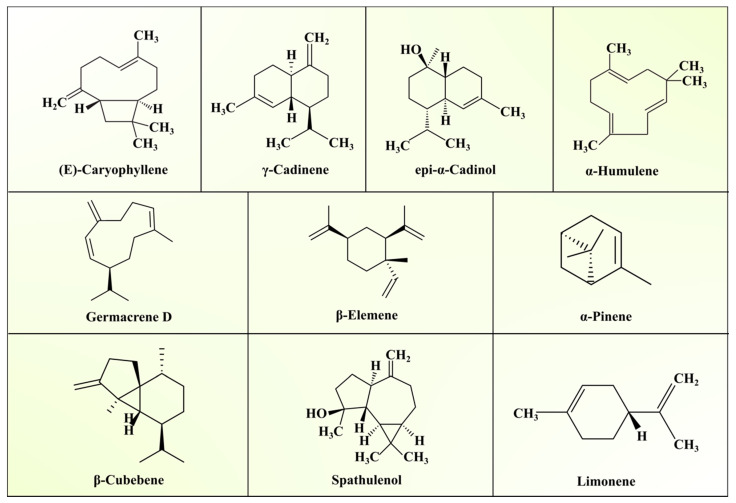
Major essential oil components in *Annona squamosa* L. leaves.

*Annona squamosa* has been widely grown in India for its fruits, apart from its leaves, stem, and roots, which are also important because they possess nutraceutical and pharmaceutical value. Furthermore, the essential oil extracted from ASLs displays excellent antiparasitic and antimalarial activity [28]. Various studies have been conducted to investigate the chemical composition of ASL essential oil (ASLEO). Shade-dried leaves collected from lower regions of the Himalayas yielded 0.13% essential oil through the hydrodistillation method. Altogether, 43 constituents were detected, which represented 88.6% of the total oil extracted from ASLs. Gas chromatography–mass spectrometry (GC-MS) and GC–flame ionisation detection (GC-FID) analyses of ASLEO showed a dominance of sesquiterpenoids (sesquiterpene hydrocarbons, 63.4%, and oxygenated sesquiterpenes, 21.8%) followed by monoterpenes (monoterpene hydrocarbons, 2.0%, and oxygenated monoterpenes, 1.4%). Among the sesquiterpenes, the major constituents were (E)-caryophyllene (15.9%), γ-cadinene (11.2%), and epi-α-cadinol (9.4%) [29]. ASLEO composition has also been studied in different parts of the world. (E)-Caryophyllene (27.4%) and germacrene D (17.1%) were major constituents reported in Brazilian ASLEO [28]; carvone (24.9%), diacetyl (9.3%), and linalool (7.7%) were major constituents reported in Egyptian ASLEO [30]; and leaf essential oil from the closely related plant *Annona muricata* from France contained germacrene D (15.7%), β-elemene (12.0%), sabinene (8.8%), and α-pinene/β-pinene (8.1%) as major components [31]. α-Pinene (1.0–11.9%), limonene (0.8–11.7%), and β-caryophyllene (11.6–24.5%) were the major constituents of ASLEO derived from Vietnam [32]. ASLEO is mainly composed of terpenes and sesquiterpenes. It yields approximately 59 chemical compounds, of which β-caryophyllene (31.1%) and δ-cadinene (6.7%) were found to be the major compounds [33]. Fresh leaves of *Annona squamosa* yielded 0.12% (*v*/*w*) essential oil through the hydrodistillation method. Eighteen constituents were identified from the ASLEO by using GC and GC/MS, which represented 86% of the total oil extracted. The northern region of India demonstrates a prevalence of β-caryophyllene (23%) and germacrene D (21.3%), while the major components of ASLEO from the southern region of India are β-cedrene (23.3%) and β-caryophyllene (14.1%) [34]. The nutritional and essential oil composition of ASLs is presented in Table 1, and Table 2 shows the various essential oil components reported by the scientific community. The structures of essential oil component present in ASLs are depicted in Figure 2.

### 3.2. Secondary Metabolite Profile

**Figure 3:**
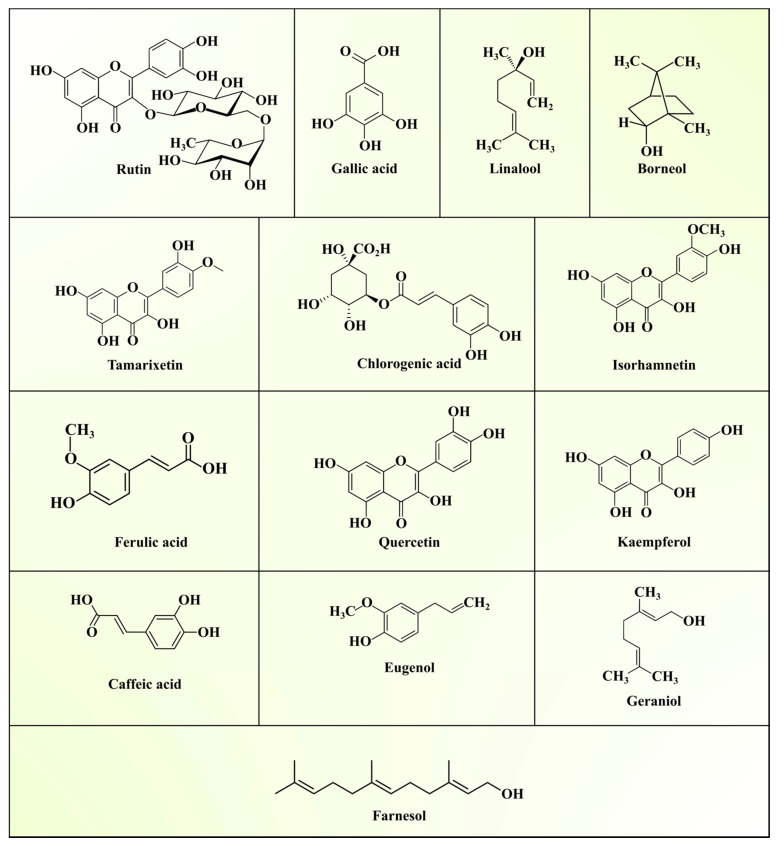
Major phytochemicals from *Annona squamosa* L. leaves.

ASLs are mostly discarded or burnt, as they are considered a worthless agricultural waste product. However, ASLs have been commonly used in tropical and subtropical countries such as India, Vietnam, Malaysia, and Laos as a traditional medicine for the treatment of cardiac problems, dysentery, worm infections, fever, fainting, haemorrhage, constipation, and dysuria. Bioactive phytochemicals investigated in different ASL extracts revealed potential biological and pharmacological activities, such as antidiabetic, antioxidant, antimicrobial, antiviral, antiobesity, antidiarrhoeal, and antitumour activities [41,42]. The phytochemical profile of ASLs can be broadly classified as acetogenins, alkaloids, flavonoids, phenols, saponins, tannins, glycosides, sesquiterpenes, anthocyanins, steroids, diterpenes, terpenoids, quinones, amino acids, and fatty acids [43,44,45,46]. Among them, total phenolic compounds (TPCs), including flavonoids, alkaloids, phenols, saponins, and tannins, are most abundant in ASLs. Epidemiological investigations have proven the role of polyphenolic compounds against various chronic diseases, such as cancer, diabetes, cardiovascular, and neurodegenerative diseases [35]. Polyphenolic compounds regulate various biochemical and physiological processes, namely, enzymatic activity, cell proliferation, signal transduction pathways, and cellular redox potential, to fight against chronic pathologies [47]. A list of various phytochemicals reported in ASLs is presented in Table 3, and the structures are depicted in Figure 3.

In recent years, custard apple cultivation has been gaining much public attention due to the essential oil extracted from its leaves. Kulkarni et al. [60] investigated the chemical composition of ASL extracts using the gas chromatography–mass spectroscopy (GC-MS) technique. They reported sodium benzoate (27.50%), 4,4-dimethylcholertrol (10.30%), 4,4-tert-butylcalix(4)areve (12.34%), stigmasterol acetate (2.92), butyloctylpthalate (9.67%), and isoamylacetyate (2.29%) in ASL extracts. Garg and Gupta [34] identified 18 compounds from essential oil extracted from ASLs collected from North Indian Plains, which account for 86% of the oil. The major constituents of ASLs oil categorised as β-Caryophyllene (23.0%), germacrene D (21.3%), monoterpene hydrocarbons (2.5%), sesquiterpene hydrocarbons (76.0%) and oxygenated sesquiterpenes (7.1%), β-elemene (7.8%), and bicyclogermacrene (8.5%). Hydro-distillation of ASLs produced red essential oils, with a yield of 0.76% (*w*/*w*), and about 95.5% of oil is contributed by sesquiterpenes [61]. In this study, 23 compounds were reported in ASLEO using GC-flame ionisation detection (GC-FID) and GC-MS. Among all, (E)-caryophyllene (27.4%), bicyclogermacrene (10.8%), germacrene D (17.1%), β-elemene (6.2%), (Z)-caryophyllene (7.3%), epi-α-cadinol (4.3%), γ-cadinene (4.2%), δ-elemene (4.1%), and α-humulene (5.7%) were found in higher concentrations.

Kumar et al. [43] reported the total phenolic content, with a range of 212.8–1478.4 μg/g, in leaf extracts of 30 different *Annona squamosa* genotypes. A total of seven phenolic compounds, including chlorogenic acid (1.84–5 μg/g), quercetin (0.19–1.60 μg/g), gallic acid (0.45–0.89 μg/g), caffeic acid (0.07–2.57 μg/g), ferullic acid (0.72–2.82 μg/g), cinnamic acid (0.02–0.05 μg/g), and salicylic acid (0.64 μg/g), were detected and quantified from the methanolic extract of ASLs. Similarly, various phytochemicals, such as phenols, anthroquinones, saponins, tannins, glycosides, and flavonoids, were reported by Katole et al. [45] in ASL extracts. In another study, Malik et al. [46] isolated and characterised rutin (quercetin-3-rhamnosyl glucoside), a flavonoid compound, from ethanolic extracts of ASLs by nuclear magnetic resonance (NMR) and MS techniques. In a recent study of ASL extracts, Nguyen et al. [42] reported flavonoid and total phenolic contents of 82.61 ± 0.82 mg of quercetin equivalent (QE)/g and 242.88 ± 6.13 mg of gallic acid equivalent (GAE)/g, respectively. These studies suggested that ASLs may serve as potential food supplements to improve human health.

## 4. Biological Activities of *Annona squamosa* L. Leaves

### 4.1. Anticancer Activity

The ability to evade apoptosis is a unique property of human cancers that can result in effective cancer progression and tumour formation. The high resistance of cancer cells to apoptosis against a pertinent stimulus is a critical rationale underlying therapy failure. Hence, a number of cancer treatment strategies, including radiation therapy and chemotherapy, are primarily based on cancer cell apoptosis [38]. A long history of using natural products as ethnomedicines, which are inexpensive and have minimal side effects, in contrast to exorbitant synthetic drugs with deleterious effects, has instigated the development of natural pharmaceutical drugs [39]. In recent decades, naturally derived bioactive compounds with apoptosis-inducing effects have garnered significant interest in the area of anticancer pharmaceuticals.

Leaves of *Annona squamosa* have a number of chemical compounds belonging to diverse groups, including phenolics, annonaceous acetogenins, saponins, flavonoids, alkaloids, glycosides, alkaloids, steroids, and terpenoids [62]. A study was conducted to investigate the in vivo and in vitro anti-breast cancer activity of ASL extracts [40]. Leaf extract at a concentration of 100 µg/mL exhibited strong cytotoxic and antiproliferative activities against breast cancer cell lines (MCF-7 and MDA-MB-231). The extracts showed a persistent induction of apoptosis and reduction in wound closure. The authors indicated that the anticancer effects could result from the cell cycle arrest of MCF-7 cells at G1 phase and inhibition of their migration to other parts of the body [59]. The inhibition of these cell lines could also be attributable to reactive oxygen species (ROS) generation, DNA fragmentation, and nuclear condensation, resulting from the downregulation of the ratio of Bcl-2 (apoptosis regulator) and Bax (induce apoptosis by instigating the release of mitochondrial cytochrome C). Additionally, the activation of Caspase-3 stimulates the expression of a number of microRNA species that target Bcl-2.

In another study, silver nanoparticles were synthesised by ASL extract (As-Ag NPs) and tested for their anticancer activity against the HeLa (cervical) cancer cell line [58]. The results indicated that these As-Ag NPs exhibited strong apoptotic action against HeLa cancer cells. This could result from atypical protein signalling and ROS induction, causing oxidative stress and subsequently inducing apoptosis and cell death [63]. A similar study was conducted to test the efficacy of ethanolic ASLs extract integrated with chitosan nanoparticles against human colon cancer cell lines (WiDr) [56]. The IC50 value against WiDr cells was estimated to be 292.39 µg/mL. The extracts were found to significantly increase the expression of caspase-3, Bax and bad genes, causing an arrested cell cycle at the G2/M phase and induction of WiDr apoptosis.

### 4.2. Antidiabetic Activity

Diabetes is an endocrine and metabolic disorder that is primarily characterised by insulin deficiency, insulin resistance, and elevated levels of sugar in the blood. According to the International Diabetes Federation (IDF), the prevalence of diabetes mellitus (DM) is escalating across the globe. In 2016, 415 million people had diabetes, and by 2040, 642 million people are anticipated to suffer from DM [57]. For the treatment of DM, numerous oral hypoglycaemic medicinal compounds, such as sulfonylurea (glimepiride), thiazolidinedione (rosiglitazone), and biguanides, are available in addition to insulin. However, counterfeit medicinal compounds have led to critical complications in patients and are harmful to pregnant women [64]. In the distant past, medicinal plants and their extracts were used to control diabetes because of their potential to ameliorate hyperglycaemia without any side effects [65]. Following the WHO guidelines on DM, investigations into medicinal plants as hypoglycaemic agents have become more relevant [66].

The antidiabetic or antihyperglycaemic effects of ASLs and their constituents have been studied in animal models. Panda and Kar [49] isolated quercetin-3-O-glucoside (Q3G) from ASL, evaluated its antidiabetic potential in alloxan-induced diabetic rats, and observed that Q3G significantly improved insulin secretion and reduced the level of glucose in the blood. The authors also observed an increase in antioxidant enzyme activity, such as superoxide dismutase (SOD) and catalase (CAT), in renal and hepatic cells. Ranjana and Tripathi [53] studied the antidiabetic properties of different extracts of ASL and found that among the methanol and water extracts of ASL, hexane extract showed a better antihyperglycaemic response. Authors reported that hexane extract (400 mg/kg) showed significant intestinal α-glucosidase inhibition with 75.69 ± 1.7% compared to acarbose (10 mg/kg) with 53.60 ± 1.45% and improved the serum insulin level with 16.26 ± 1.20 µU/mL compared to glimepiride (1 mg/kg) with 17.36 ± 0.90 µU/mL, which demonstrated the secretagogue nature of the extract. Davis et al. [51] implied that leaves exert their action by promoting glucose uptake as well as by modulating insulin signalling events via inhibition of protein tyrosine phosphatase 1B (PTP1B) and stimulation of the phosphorylation of insulin receptor-β (IR-β) and insulin receptor substrate-1 (IRS-1) accompanied by upregulation of glucose transporter type 4 (GLUT4) and phosphoinositide 3-kinase (PI3 kinase) mRNA expression in vitro. The authors also revealed that oral administration of hexane extract showed a significant reduction of 30.5% in the levels of triglyceride (TG) compared to the antidiabetic drug rosiglitazone (33.5%). Gupta et al. [64] evaluated the effects of an ethanol extract of ASLs in alloxan and streptozotocin (STZ)-induced diabetic mice. The authors determined that a dose of 350 mg/kg body weight significantly decreased the fasting blood glucose (FBG) level by 26.8%, reduced the triglyceride (TG) by 28.7%, significantly reduced the low-density lipoprotein (LDL) or “bad cholesterol” by 71.9% along with total cholesterol (TC) by 49.3%, and favourably increased high-density lipoprotein (HDL) or “good cholesterol” by 30.3% in diabetic mice. In several studies, researchers investigated the hypoglycaemic and antidiabetic effects of ASLs and reported that these extracts exerted antidiabetic potential through (i) increasing the amount of insulin by stimulating pancreatic β-cells, (ii) increasing glucose uptake in the muscle, (iii) inhibiting intestinal enzymes such as α-glucosidase responsible for glucose metabolism [66], and (iv) inhibiting glucose output from the liver and consequently improving the lipid profile in diabetic mice [52,54]. The abovementioned findings suggested that ASLs could be useful to maintain or control normal blood sugar levels and cholesterol levels and could be an effective alternative as a herbal medicine to control diabetes. Antidiabetic and various other bioactivities of ASL extract is presented in Table 4. 

### 4.3. Antioxidant Activity

In the course of metabolic reactions, cells produce free radicals as a byproduct. Free radicals induce oxidative stress in the cell at higher concentrations, which contributes to cell function disruption. Multiple recent studies have shown that deleterious free radicals cause various degenerative diseases, such as diabetes, cancer, and neurodegeneration [80]. Several studies have shown the significance of antioxidant compounds from ASLs for mitigating the damaging effects of free radicals [82]. Gas chromatography coupled with mass spectrometry (GC-MS) analysis of the methanolic extract of ASLs revealed the presence of sesquiterpene hydrocarbons, such as germacrene-D (22.01%), trans-caryophyllene (12.12%), and bicyclogermacrene (2.80%) [18]. Another such preliminary phytochemical screening of ASL extract revealed the presence of alkaloids, flavonoids, phenols, and saponins [42]. Several studies were conducted to determine the antioxidant potential of the ASLs. The DPPH (2,2-diphenyl-1-picrylhydrazyl) scavenging activities of ASL extracts in acetone, methanol, and water were reported to have moderate antioxidant activity with an IC50 value of 33.9 ± 4.8 μg/mL for acetone, followed by methanol and aqueous extracts with values of 51 ± 1.6 and 98.3 ± 0.4 μg/mL, respectively [18]. Another such study of ASL extracts in methanol and ethanol resulted in IC50 = 13.61 µg/mL and IC50 = 15.97 µg/mL, respectively [72]. The DPPH free radical scavenging activity of ASL extracts in chloroform, methanol, and water reported IC50 values of 308.3 μg/mL, 342.5 μg/mL, and 439.6 μg/mL, respectively. The NO radical scavenging activity of the extracts reported IC50 values of 185.2 mg/mL, 345.8 mg/mL, and 366.3 mg/mL in methanol, chloroform, and water, respectively. The overall study reported that the methanol extract had higher antioxidant activity than chloroform and water, which was attributed to the presence of a large class of phytochemicals that were absent in chloroform and water extracts. The study also reported phenolic compounds as the main antioxidant components [1]. Another study reported the H_2_O_2_ scavenging activity of different solvent extracts of ASL in methanol, acetone, ethanol, and water, where water extracts exhibited higher activity, i.e., 54.06%, at 25 μg/mL than acetone, ethanol, and methanol extracts. The study also reported the correlation coefficient between the total antioxidant activity and total phenolics, which was found to be 0.8965 [3]. A previous study attributed the difference in H_2_O_2_ scavenging capacity between the solvent extracts to the structural features of their active components, which determine their electron-donating abilities [73]. In another study ethanolic extract of ASL was reported to have 133.33 and 264.65 μg/mL DPPH and hydroxyl activity, respectively [83]. The authors concluded that polyphenols extracted from the ASL at optimised conditions have excellent antioxidant properties which can be utilised in preparation of functional foods. In a parallel comparative study, methanol and hexane extract of ASL reported IC50 49.64 and 64.01 µg/mL, respectively [84]. The authors also showed that ASL extracts have superior antioxidant properties than bark extracts. In summary, many interesting results indicated the potential of ASL extracts as antioxidants, but little research on the application of these extracts has been reported.

### 4.4. Antimicrobial Activity

Plant-based antimicrobials have substantial prophylactic properties and are considered to be efficient, safe, and cost-effective alternatives for synthetic antimicrobials that possess more notable side effects. Despite being a commercial fruit plant because of its creamy succulent flesh, *Annona squamosa* is reported to have enormous pharmacological properties, including antimicrobial activity, owing to the presence of different secondary metabolites, such as glycosides, phytosterols, alkaloids, oils, saponins, phenols, and flavonoids [85]. In several research studies, the leaf extracts of *Annona squamosa* were identified to have remarkable antibacterial activity and antifungal activities. Significant antimicrobial activities were exhibited by an active acetogenin compound known as annotemoyin isolated from chloroform leaf extract and by certain flavonoid compounds purified from aqueous leaf extract of the plant [44]. Other acetogenins, such as squamocin, squamostatin, and cholesteryl glucopyranoside, showed growth inhibition of gram-positive bacteria, such as Bacillus subtilis, Bacillus cereus, Bacillus megaterium, and Staphylococcus aureus [74]. LC-MS/MS analysis of ASL extracts from plants growing in drylands identified the presence of metabolites, including ephedradine A, ergosine, mudanpioside H, and trichosanic acid, exhibiting significant antifungal activity. The same extract was found to inhibit bacterial growth at a concentration of 8–11 µg, and the compound mudanpioside H was possibly responsible for this antibacterial activity [81]. Additionally, screening for comparative antimicrobial activity of methanol and petroleum ether leaf extracts of *Annona squamosa* was performed against two gram-positive *(S. aureus* and *B. subtilis*) and two gram-negative (*Escherichia coli* and *Pseudomonas aeruginosa*) bacteria, indicating that the highest zone of inhibition was observed against *P. aeruginosa* with a minimum inhibitory concentration (MIC) of 131 µg/mL, followed by petroleum ether extract against P. aeruginosa with an MIC of 164 µg/mL and methanol extract against *E. coli* with an MIC of 179 µg/mL. Here, phytochemical studies proved that some of the essential oil compounds, such as linalool, borneol, eugenol, farnesol, and geraniol, present in the leaf extracts contributed to the antibacterial activity [77].

The natural metabolite palmitone purified from leaf cuticular wax of *Annona squamosa* showed higher antibacterial activity (MIC: 6.24–12.6 µg/mL) than its individual isomeric hydroxyl ketones, i.e., 11-hydroxy-16-hentriacontanone and 10-hydroxy-16-hentriacontanone (MIC: 20–52 µg/mL). For a compound to penetrate the bacterial cell wall, the lipophilicity of the cell wall and the specific compound need to be mutually compatible. Palmitone is a symmetrical ketone compound compared to hydroxy palmitones, and therefore, has a greater penetration ability and higher antimicrobial activity [86]. Additionally, antiviral activity was exhibited by kaurane diterpenoid 16β,17-dihydroxy-entkauran-19-oic acid, which inhibited HIV replication in H9 lymphocyte cells [78]. The antimicrobial mechanism of action of leaf extracts can be attributed to the major contributors, i.e., phenolic compounds, which induce disruption of the microbial membrane, coagulation of cytoplasmic components, and cytoplasmic leakage, interfering with microbial cellular metabolism and adapting anti-quorum sensing activity [79]. A study on the prospective role and applications of ASL extract in the prevention of foodborne bacterial disease indicates that the extract has broad-spectrum but heat-labile activity against foodborne bacterial pathogens because of its ability to scavenge H_2_O_2_ in a range of 45–55% [87]. Detailed research should be performed to explore the myriad of bioactive compounds that combine to give the leaf extract its therapeutic value, their mechanisms of action and more potential benefits of using ASLs as natural food preservatives.

### 4.5. Hepatoprotective Properties

Hepatosteatosis or fatty liver disease (FLD) is mostly caused by an imbalance in the production and metabolisation of fat in the body, which is caused by dietary habits, sedentary lifestyles, and stress. Moreover, several drugs (e.g., paracetamol) are known to cause damage (as a side effect) to hepatocytes. Due to the effect of a high fat dose and the damage caused by drugs, hepatocytes face oxidative stress, which further leads to hepatosteatosis. Drug-induced liver injury has become a major concern for health-related personnel and industry [69]. Therefore, compounds that act as antioxidants, inhibitors of lipid peroxidation, and possess free radical scavenging capacity may show hepatoprotective properties. ASLs exhibit very high antioxidant activity (Section 4.3). The presence of flavonoids, glycosides, saponins, alkaloids, and phenolic compounds was reported by Rajeshkumar et al. [71] in ASLs. Paracetamol is the most common medicine used in the treatment of various diseases, but it has many negative impacts on various body parts. Consumption of paracetamol causes hyperlipidaemia, which is a common sign of liver damage. Rajeshkumar et al. [71] used paracetamol-treated (2 g/kg b.w.) albino rats to evaluate the hepatoprotective properties of ASLs. The dose of ASL extract (1000 mg/kg body weight (b.w.)) conferred effective protection of hepatic cells by enhancing protein levels and inducing a significant reduction in serum glutamate oxaloacetate transaminase and serum glutamate pyruvate transaminase levels in serum. The presence of a flavone [5,7,40-trihydroxy-6,30 dimethoxy flavone 3-O-a-L-rhamnopyranoside (THDMF-Rha)] was reported in the ASL extract. At a dose of 5.0 mg/kg b.w., THDMF significantly reduced hepatic lipid peroxidation in L-thyroxine-induced rats [48]. In another individual experiment, the hepatoprotective properties of ASLs were evaluated in carbon tetrachloride-induced Wistar rats by Sonkar et al. [67]. These rats were further treated with ethanolic extract of ASLs (450 mg/kg b.w.), which reduced malondialdehyde (MDA) levels (45.48%), total serum bilirubin (38.82%), alanine aminotransferase (39.51%), and aspartate aminotransferase (11.69%). Moreover, the effect was similar to that of a hepatoprotective compound (silymarin) after 7 days of treatment. The authors speculated that the presence of coumarins may be the chief factor conferring hepatoprotective activity. A significant improvement was observed in islands of Langerhans in rats treated with aqueous and ethanolic extracts at a dose of 300 mg/kg/day [70]. In the case of diethylnitrosamine (200 mg/kg)-induced swiss albino mice, the levels of glutamyl oxaloacetate transaminase (GOT), glutamyl pyruvate transaminase (GPT), alkaline phosphatase (ALP), acid phosphatase (ACP), alpha fetoprotein (AFP), and total bilirubin increased in serum and tissue, and these were significantly reduced following treatment with ASL extract (5 g/kg for 30 days) [69]. Upon application at 250 and 500 mg/kg b.w., the ASL methanolic extract significantly decreased the serum concentrations of bilirubin, ALP, aspartate amino transferase (AST), gamma glutamate transpeptidase (γ-GT), alanine amino transferase (ALT), and TBARS, but increased the total protein concentration in rifampicin-induced male Wistar albino rats [88]. These results demonstrated that ASL extracts can be used as hepatoprotective agents and to improve the condition of hepatocytes.

### 4.6. Effect of ASLs Extract on the Lipid Profile

A balanced lipid profile or lipid panel is required for the proper metabolic function of the body. An imbalance in the lipid profile (dyslipidaemia) leads to several diseases, such as coronary artery disease, stroke, peripheral arterial disease, cancer, atherosclerosis, and myocardial infarction. Lifestyle, food habits, disease, and drugs are responsible for dyslipidaemia due to the enhanced mobilisation of lipids from adipose tissue to plasma. Diabetes, one of the diseases that affects antioxidant defence systems, causes oxidative stress and alters the lipid profile due to overproduction and/or insufficient removal of ROS [52,70]. Diabetic Wistar rats and albino rabbits were treated with ethanolic extract of ASLs at various doses (200, 300, 350, and 400 mg/kg). The most significantly positive result was observed with a dose of 350 mg/kg, which reduced TC, LDL, and TGs by approximately 49.3%, 71.9%, and 28.7%, respectively, and increased the level of HDL (30.3%) [64]. In another experiment, streptozotocin (50 mg/kg) induced diabetic albino Wistar rats treated with a water extract of ASLs. Treatment reduced MDA levels, activated antioxidant enzymes, and maintained the lipid profile (lower TGs, TC, and higher HDL) [52]. Diabetic mice treated with methanolic extract of ASLs (250 mg/kg body weight) significantly reduced serum TC (34.7%), TGs (50%), and LDL (53%) and improved HDL (53%) levels compared to nontreated mice. The author suggests that the activation of lipoprotein lipase (LPL) and stimulation of β-cells are responsible for hypolipidaemic activity, as they secrete sufficient insulin to clear triglycerides from plasma [75]. The application of an alcoholic extract of ASLs in streptozotocin-induced albino rats significantly reduced TC and TGs but increased HDL. Moreover, with the improvement of the lipid profile, a significant weight reduction (19.46%) was reported in the treated rats compared to the control after 28 days of treatment [76]. The presence of higher levels of flavonoids in the leaf may be responsible for the same result. Methanolic extract containing THDMF-Rha was found to be effective in reducing lipid concentrations (TC, TGs, VLDL, LDL, and HDL) in serum [48]. In another study, the application of both aqueous and ethanolic ASL extracts (300 mg/kg b.w.) significantly decreased the levels of CL, TGs, and LDL cholesterol and significantly increased HDL cholesterol levels in alloxan (150 mg/kg)-induced diabetic rats [70]. The present study has shown that ASL extracts potently cause weight loss and maintain the lipid profile in serum.

Extracts from the natural sources are rich in bioactive compounds and perform numerous biological activities, which is key for the formulation of pharmaceutical drugs and innovative food products rich in antioxidants. ASLs have been grossly studied for their health promoting effects using in vivo ad in vitro experiments. However, to establish ASLs as an active ingredient in pharma- and food industry, more clinical trials must be conducted. Furthermore, ASL extracts have been proven to be cytotoxic against cancerous cells, but its toxicity needs more exhaustive studies on the normal cells. Vast molecular diversity of the ASLs have potential to treat other ailments in humans, which needs further attention of the researchers.

## 5. Conclusions

ASLs exhibit excellent nutraceutical, phytochemical, biological, and pharmacological activities due to the presence of distinct metabolites, phenolic compounds, flavonoids, and other active components. ASLs offer great advantages in terms of being a natural fruit ingredient, nutritional food component, and natural medicinal plant that has been utilised all over the globe for the treatment of numerous acute and chronic ailments, such as diabetes, cardiac, cancer, and immune-related disorders. ASLs offer high nutritional value, consisting of protein, fibre, carbohydrates, and vitamins, for both humans and animals, thus making them a complete food and natural medicinal agent. Furthermore, ASLs contain high amounts of sesquiterpenoids and terpenes, and the chemical profile of ASL shows 43 different compounds that mainly give rise to essential oil. Various phytochemicals present in ASLs include glycosides, phytosterols, proteins, carbohydrates, saponins, tannins, alkaloids, phenols, flavonoids, peptides, and acetogenins. These compounds contribute to numerous bioactivities of ASL extracts, including anticancer, antidiabetic, antioxidant, hepatoprotective, antimicrobial, and lipid-lowering effects. Hence, ASLs are potential cost-effective ingredients for nutraceutical, medicinal, and food applications. The phytochemical and pharmacological activities of ASLs make them an indispensable and essential component for natural medicine, immunity boosters, and health-promoting ingredients. Many studies have considered phytochemical profiles, but few studies are available on the carbohydrates, proteins, and lipids present in ASLs. Exploration of the molecular mechanisms underlying the various bioactivities contributed by ASLs is also necessary. Furthermore, there is a need to develop value-added products from ASLs to improve their utilisation as health-promoting ingredients.

## Figures and Tables

**Table 1:** Nutritional and essential oil component composition of *Annona squamosa* L. leaves.

Group	Composition	References
Protein	mg/g of FW (fresh weight)	[27]
Egyptian sites		
Menofia	13.47 ± 0.11	
Giza	6.80 ± 0.11	
Alexandria	3.52 ± 0.10	
Mansoura	17.26 ± 0.02	
Essential oil profile		
North Indian (Foothills region) ASLEO	Composition (%)	[35]
(E)-Caryophyllene	15.9	
γ-Cadinene	11.2	
epi-α-Cadinol	9.4	
(Z)-Caryophyllene	7.3	
γ-Muurolene	5.4	
α-Humulene	5.2	
North Indian (Plains) ASLEO		[36]
β-Caryophyllene	22.9	
Germacrene D	21.3	
Bicyclogermacrene	8.5	
β-Elemene	7.8	
γ-Cadinene	6.7	
α-Muurolol	5.7	
Vietnamese ASLEO		[37]
α-Pinene	1.0–11.9	
Limonene	0.8–11.7	
β-Cubebene	0.5–13.0	
β-Caryophyllene	11.6–24.5	
Spathulenol	0.8–9.0	
Caryophyllene oxide	1.0–10.6	
α-Cadinol	3.3–7.8	
Brazilian ALEO		[38]
(E)-Caryophyllene	27.4	
Germacrene D	17.1	
Bicyclogermacrene	10.8	
(Z)-Caryophyllene	7.3	
β-Elemene	6.2	
δ-Elemene	4.1	

**Table 2:** Essential oil components reported in *Annona squamosa* L. leaves.

Variety	Type of Extract	Essential Oil Components Identified	References
ASLs, Lucknow, India	Hydro-distilled essential oil	Limonene, terpinolene, bicyclogermacrene, γ-cadinene, α-copaene, α-muurolol, β-bourbonene, δ-cadinene, (Z)-nerolidol, β-elemene, β-caryophyllene, (E)-nerolidol, caryophyllene oxide, γ-elemene, aromadendrene, γ-eudesmol, germacrene D, α-humulene	[36]
ASLs	Methanol, petroleum ether, chloroform, and water extracts	Linalool, borneol, eugenol, farnesol, geraniol	[39]
ASLs, city of Sao Cristovao, Sergipe, Brazil	Hydro-distilled essential oil using a Clevenger-type apparatus	α-Pinene, camphene, limonene, δ-elemene, α-copaene, β-bourbonene, β-elemene, (*Z*)-caryophyllene, (*E*)-caryophyllene, α-humulene, germacrene D, viridiflorene, bicyclogermacrene, germacrene A, γ-cadinene, δ- cadinene, germacrene B, spathulenol, caryophyllene oxide, *epi*-α-cadinol, α-cadinol	[40]

**Table 3:** Secondary metabolite profile of *Annona squamosa* L. leaves.

Variety	Type of Extract	Bioactive Compounds Identified	References
ASLs, Diliman, Quezon City, Philippines	95% ethanol extract	Acetogenin murihexocin C	[48]
ASLs	Ethanol extract	Stigmasterol acetate, 4,4-tert-butylcalix(4)areve, sodium benzoate, 4,4-dimethylcholertrol, isoamylacetyate, butyloctylpthalate	[49]
ASLs, Mayurbhanj, Orissa, India	Water, petroleum ether, and methanol extracts	Linalool, flavonoids, eugenol, borneol, geraniol, farnesol	[50]
ASLs, Fortaleza, State of Ceara, Brazil	80% methanol extract	O-methylarmepavine, C37 trihydroxy adjacent bistetrahydrofuran acetogenins	[51]
ASLs, Chennai, Tamilnadu, India	Ethanol extract	Rutin, kamepherol, quercetin, isorhamnetin, farmarixetin	[52]
ASLs, Fukuoka, Japan	Methanol extract	Lanuginosine, liriodenine, lysicamine	[53]
ASLs	Methanol extract	5,7,4′-trihydroxy-6,3′dimethoxy-flavone 5-O-α-L-rhamnopyranoside	[54]
ASLs, Dharwad, Karnataka, India	Water, methanol, and chloroform extracts	Phenols, glycosides, flavonoids, saponins, tannins, alkaloids, steroids, and carbohydrates	[1]
ASLs, Kattakada, Thiruvananthapuram, Kerala, India	Water, acetone, and chloroform extracts	Alkaloids, glycosides, saponins, oils, and flavanoids	[55]
ASLs, Pune, India	Water and methanol extracts	Saponins, tannins, anthroquinones, phenols, flavonoids, and glycosides	[56]
ASLs, Bhopal, Madhya Pradesh, India	80% ethanol extract	Rutin (quercetin-3-rhamnosyl glucoside)	[57]
ASLs, Junagadh, Gujarat, India	Methanol extract	Gallic acid, quercetin, chlorogenic, cinnamic acid, ferullic acid, caffeic acid, and salicylic acid	[58]
ASLs, Duyen Hai, Tra Vinh, Vietnam	Water and ethanol extracts	Alkaloids, saponins, coumarins, flavonoids, cardiac glycosides, phenols, and tannins	[59]

**Table 4:** Biological activities of *Annona squamosa* L. leave extracts.

Variety	Type of Extract	Bioactive Compounds Identified	Type of Cell Lines/Type of Study	Major Findings and Molecular Mechanisms of Action	References
Anti-cancer activities					
Leaves were obtained from a local plant nursery in Ta’if City, Saudi Arabia	Methanolic and acetonic extracts	Phenolics, annonaceous acetogenins, saponins, flavonoids, alkaloids, glycosides, alkaloids, steroids, and terpenoids	MCF-7 and MDA-MB-231 breast cancer cell lines	100 µg/mL extract decreases cell viability and reduced their proliferation to ~60%.	[62]
Leaves were obtained from botanical garden in Narsapur, W.G.Dt, South India	Extracts prepared using double distilled water	NA	HeLa (cervical) cancer cell line	IC50 value against HeLa cells was estimated to be 25 μg/mL.	[67]
Leaves were obtained from Lumajang Regency, East Java, Indonesia.	Ethanolic extract	12,15-cis-squamostatin-A, bullatacin	Human colon cancer cell lines (WiDr)	IC50 value against WiDr cells was estimated to be 292.39 µg/mL.	[68]
Leaves were obtained from department of Pharmacognosy, Faculty of Pharmacy, University of Karachi, Pakistan	Ethanolic extract	Annoreticuin and Isoannoreticuin	Colon cancer cell line (HCT-116), breast carcinoma cell line (MDA-MB-435), prostatic cancer cell line (DU145), human epidermoid carcinoma cell line (KB-3-1), lung cancer line (H460), and hepatocellular carcinoma cell line (BEL7404)	IC50 values of 13.66 µg/mL for KB 3-1 cells, 1.37 µg/mL for HCT-116 cells, 74.51 µg/mL for HEK293 cells, 53.86 µg/mL for KB-3-1 cells, and 15.06 µg/mL for HCT-116 cells.	[55]
Anti-diabetic activity					
ASLs were collected from IARI, New Delhi, India	Ethanolic extract		In vivo (Wistar strain of rats with alloxan (80 mg/kg) and STZ (50 mg/kg i.p.) induced diabetes)	Administration of ASLs extract (350 mg/kg), significantly reduced the FBG by 6%, 26.8%, and 13% in normal, alloxan-induced, and STZ induces diabetic rats, respectively, and improved the glucose tolerance in diabetic rats. ASL also reduced TC (by 49.3%), LDL (by 71.9%), and TG (by 28.7%) and increased HDL (by 30.3%) in severely diabetic mouse.	[69]
Young ASLs were collected from Painkulam village, Tamil Nadu, India.	95% Ethanol extract		In vivo (Wister male albino rats with STZ (65 mg/kg i.p.) induced diabetes)	Administration of ASLs extract (250 mg/kg and 500 mg/kg) exhibited a significant reduction of FBG and increased the insulin level.	[70]
ASLs	Hexane extract		In vitro (L6 Myotubes) and in vivo (Ob/ob mice modal)	ASLs hexane extract (500 mg/kg b.i.d. p.o.) improved the glucose uptake, stimulated the IR-β and IRS-1 phosphorylation, and promoted the upregulation of mRNA (GLUT4 and PI3 kinase) in L6 myotubes. ASLs hexane extract inhibited the PTP1B with an IC50 17.4 µg/mL Oral administration of ASL hexane extract significantly declined random glucose (27.7%) and TG (30.5%).	[51]
ASLs	Water extract, Hexane extract, and methanol extract		In vivo (CF strain rats)	Hexane extract inhibited the α-glucosidase (75.69 ± 1.7%) in comparison to acarbose (53.60 ± 1.45%) Hexane extract (100 mg/kg and 400 mg/kg) improved insulin level (11.58 ± 1.8 µU/mL and 16.28 ± 1.2 µU/mL) and reduced BGL (41.18 ± 2.46% and 78.10 ± 1.57%), respectively.	[52]
Young ASLs were collected from the Regional Research Institute of Unani Medicine, Aligarh, India.	Aqueous extract		In vivo (male albino Wister rats with STZ (55 mg/kg i.p.) induced diabetes)	Oral administration of ASLs extract (300 mg/kg) significantly reduced the BGL, lipid peroxidation and also increased the activity of the antioxidant enzymes.	[71]
ASLs collected from Kolli Hills, Tamil Nadu, India	95% ethanol extract		In vivo (male albino Wister rats with STZ (50 mg/kg i.p.) induced diabetes)	ASLs extract (100 mg/kg) significantly reduced BGL, glycosylated hemoglobin, creatinine, and urea.	[72]
ASLs were collected from IARI, New Delhi, India	Water extract		In vivo (Albino Wister rats with STZ (50 mg/kg i.p.) induced diabetes)	ASLs extract (350 mg/kg) significantly improved the lipid profile and increased the activities of antioxidant enzymes.	[66]
ASLs were collected locally, Indore, India.	80% Methanol	Quercetin-3-O-glucoside	In vivo (Wistar male rats with alloxan monohydrate (120 mg/kg i.p.) induced diabetes)	Quercetin-3-O-glucoside (15 mg/kg p.o.) significantly increased serum insulin, decreased glucose, reduced oxidation of lipid (*p* < 0.001), and increased antioxidant enzyme activity (*p* < 0.001).	[49]
Antioxidant activities					
ASLs, Rajshahi, Bangladesh	Methanol extracts	Phenols and flavonoids	In vitro	IC50 of 7.81 ± 0.1 μg·mL^−1^ for DPPH, IC50 of 29.60 ± 0.17 (μM of Trolox for oxygen radical absorbance capacity (ORAC).	[73]
ASLs, Tamil Nadu, India	Ethanol extracts	Flavonoids	In vitro	IC50 of DPPH, ABTS, superoxide dismutase, nitric oxide, and lipid peroxidation were found to be 110, 40, 115, 60, and 955 μg·mL^−1^, respectively.	[74]
ASLs, Andhra Pradesh, India	Ethanol extracts	Flavones	In vitro	% DPPH radical scavenging was 45.62 at 100 (μg·mL^−1^) concentration.	[60]
ASLs, Egypt	Methanol 80%, acetone 50%, ethanol 50%, and boiling water.	Phenols	In vitro	Total antioxidant activity was reported highest in acetone extract i.e., 1625.38 ± 68.55 ascorbic acid/g of extract, and lowest in case of water 639.65 ± 22.17 ascorbic acid/g of extract.	[3]
Anti-microbial activities					
Leaf Extract (India)	Control (1 mL of 2% Gum acacia)	Steroids Alkaloids Glycosides Saponin Flavonoid Tannin Triterpenoid	Antibacterial activity and measurement of wound healing activity of ASLs extract in Albino wistar rats.	Period of epithelisation—25 days	[61]
	Petroleum ether			Zone of inhibition—19–22 mm at MIC-200 mg/ 0.1 mL. On addition of 300 mg/ kg (ED50 value) petroleum ether extract, wound healing induced within 16 days.	
	Chloroform-water			Zone of inhibition- 19–21 mm at MIC-200 mg/ 0.1 mL. On addition of 300 mg/kg (ED50 value) chloroform-water extract, wound healing induced within 19 days.	
	Alcohol			Zone of inhibition- 18–20 mm at MIC-200 mg/ 0.1 mL. On addition of 300 mg/ kg alcoholic extract, wound healing induced within 18 days.	
ASLs extract (Egypt)	Methanol 80%	Carbohydrates, tannins, phenolic compounds, polyphenols, and flavonoids	Antibacterial activity of ASLs extracts using disc-diffusion method against six bacterial species.	Zone of inhibition diameter: 12–13 mm with 38–43% inhibition.	[3]
	Acetone 50%			Zone of inhibition diameter: 14–16 mm with 41–51% inhibition.	
	Ethanol 50%			Zone of inhibition diameter: 12–14 mm with 35–48% inhibition.	
	Boiling water			Zone of inhibition diameter: 9–11 mm with 28–36% inhibition.	
ASLs extract (India)	Ethanol extract 25% 50% 75% 100%	Polyphenols tannins, and terpenoids	Estimation of the effect of ASLs extract on inhibition of six bacterial species	Zone of inhibition (in mm) 8–15 mm 10–17 mm 13–19 mm 15–22 mm	[74]
Hepato-protective and lipid lowering effect					
Leaves obtained near to NBRI, Lucknow, India	Ethanolic extract		carbon tetrachloride induced Wistar rats	Significant hepatoprotective effect was reported at oral dose of 450 mg/kg. Effects were comparable to silymarin.	[75]
ASLs extract	Ethanolic extract		Diethylnitrosamine induced Swiss albino mice	Dose of 5 g/Kg exhibited hepatoprotective effects.	[76]
ASLs extract	Aqueous extracts		Albino rats	ASLs extract protect the hepatic cells, paracetamol-induced increased level of bilirubin, cholesterol, and triglycerides level, which get normalised after treatment.	[77]
ASLs extract	Methanolic extract	5,7,40-trihydroxy- 6,30dimethoxy flavone 3-O-a-L-rhamnopyranoside	Wistar albino rats	Significantly reduced hepatic lipid peroxidation and improved serum lipid profile.	[78]
ASLs collected from Madurai, TN, India	Methanolic extract	-	Isoniazid-rifampicin induced rats	ALs extract protects against rifampicin-induced oxidative liver injury.	[79]
Leafy twigs of *A. squamosa*	Methanolic extract	-	Streptozotocin induced mice diabetic models	Significantly lower levels of TC and TGs was reported compared to diabetic control mice.	[74]
Fresh ASLs collected from Al-Nobaria, Egypt	Aqueous and ethanolic extract	-	Alloxan-induced hyperglycemic rats	Significantly reduced CL, TGs, and LDL-cholesterol and increased HDL-cholesterol compared to diabetic rats.	[80]
ASLs collected near Udaipur, India	70% alcohol	-	Streptozotocin induced Albino rats	Maintained the lipid profile (reduced CL, TGs, and LDL-cholesterol and increased HDL).	[81]
ASLs were collected near IARI, New Delhi, India	Ethanolic extract	-	Alloxan and streptozotocin induced Wistar rats and albino rabbits	After 15 days of treatment, lipid profile maintains nearly normal level and increased the HDL cholesterol.	[69]
ASLs extract	Water extract	-	Streptozotocin induced Albino Wistar rats	Treatment enhanced the activity of antioxidant enzyme and reduces MDA, CL, and TGs.	[66]

FBG—fasting blood glucose; BGL—blood glucose level; STZ—streptozotocin; TC—total cholesterol; TG—triglyceride; LDL—low density lipoprotein; HDL—high density lipoprotein; IR-β—insulin receptor-β; IRS-1—insulin receptor substrate-1; GLUT 4—glucose transporter type 4; PI3 kinase—phosphoinositide 3-kinase; PTP1B—protein-tyrosine phosphatase 1B; b.i.d.—twice a day; p.o.—by mouth.
